# Neuropsychological, neuroimaging and autopsy findings of butane encephalopathy

**DOI:** 10.1186/s12883-023-03278-4

**Published:** 2023-06-09

**Authors:** Jaeseob Yun, Sung Hwan Jang, Huiran Cho, Myung Jun Lee, Na-Yeon Jung, Jae-Hyeok Lee, Jin-Hong Shin, Young Min Lee, Jin A Yoon, Kyoungjune Pak, Junkyeung Ko, Jae Meen Lee, Chungsu Hwang, Jae Woo Ahn, Suk Sung, Kyung-Un Choi, Gi Yeong Huh, Eun-Joo Kim

**Affiliations:** 1 Department of Neurology, Pusan National University Hospital, Pusan National University School of Medicine and Medical Research Institute, 179, Gudeok-ro, Seo-gu, Busan, 49241 Republic of Korea; 2 Department of Neurology, Pusan National University Yangsan Hospital, Pusan National University School of Medicine, Yangsan, Republic of Korea; 3 Research Institute for Convergence of Biomedical Science and Technology, Pusan National University Yangsan Hospital, Yangsan, Republic of Korea; 4 Department of Psychiatry, Pusan National University Hospital, Pusan National University School of Medicine, Busan, Republic of Korea; 5 Department of Rehabilitation Medicine, Biomedical Research Institute, Pusan National University School of Medicine, Pusan National University Hospital, Busan, Republic of Korea; 6 Department of Nuclear Medicine, Pusan National University Hospital, Pusan National University School of Medicine, Busan, Republic of Korea; 7 Department of Neurosurgery, Medical Research Institute, Pusan National University Hospital, Busan, Republic of Korea; 8 Department of Pathology, Pusan National University School of Medicine, Yangsan, Republic of Korea; 9 Department of Anatomy, Pusan National University School of Medicine, Yangsan, Republic of Korea; 10 Department of Forensic Medicine, Pusan National University School of Medicine, Yangsan, Republic of Korea

**Keywords:** Butane, Encephalopathy, Neuropsychology, Neuroimaging, Autopsy

## Abstract

**Background:**

Butane is an aliphatic hydrocarbon used in various commercial products. While numerous reports of sudden cardiac-related deaths from butane inhalation have been described, butane-associated acute encephalopathy has rarely been reported.

**Case presentation:**

A 38-year-old man presented with cognitive dysfunction after butane gas inhalation. Neuropsychological test results showed impairments in verbal and visual memory, and frontal executive function. Diffusion weighted MRI revealed symmetric high-signal changes in the bilateral hippocampus and globus pallidus. FDG-PET demonstrated decreased glucose metabolism in the bilateral precuneus and occipital areas and the left temporal region. At the 8-month follow-up, he showed still significant deficits in memory and frontal functions. Diffuse cortical atrophy with white matter hyperintensities and extensive glucose hypometabolism were detected on follow-up MRI and FDG-PET, respectively. Brain autopsy demonstrated necrosis and cavitary lesions in the globus pallidus.

**Conclusions:**

Only a few cases of butane encephalopathy have been reported to date. Brain lesions associated with butane encephalopathy include lesions in the bilateral thalamus, insula, putamen, and cerebellum. To the best of our knowledge, this is the first report on bilateral hippocampal and globus pallidal involvement in acute butane encephalopathy. The pathophysiology of central nervous system complications induced by butane intoxication is not yet fully understood. However, the direct toxic effects of butane or anoxic injury secondary to cardiac arrest or respiratory depression have been suggested as possible mechanisms of edematous changes in the brain after butane intoxication.

## Background

Butane is an aliphatic hydrocarbon that is commonly used as a commercial product or chemical agent (e.g., cigarette or charcoal lighter fuel, hair spray, and aerosol). Butane inhalation can cause sudden death by cardiac arrest following cardiac arrhythmia and vagal stimulation [1]. It also causes respiratory depression and hypoxia due to oxygen replacement, leading to encephalopathy [2]. While there have been several reports of fatal cardiac toxicity after butane inhalation [3–6], cases of acute encephalopathy associated with butane intoxication have rarely been reported [1, 2, 7, 8]. Furthermore, little is known about the serial neuroanatomical and functional changes and neuropsychological sequelae related to butane encephalopathy. We report a patient who presented with cognitive dysfunction after butane inhalation, with serial brain magnetic resonance imaging (MRI), [^18^ F]-fluoro-2-deoxy-D-glucose positron emission tomography (FDG-PET), neuropsychological assessment over an 8-month period, and autopsy findings.

## Case presentation

**Fig. 1:**
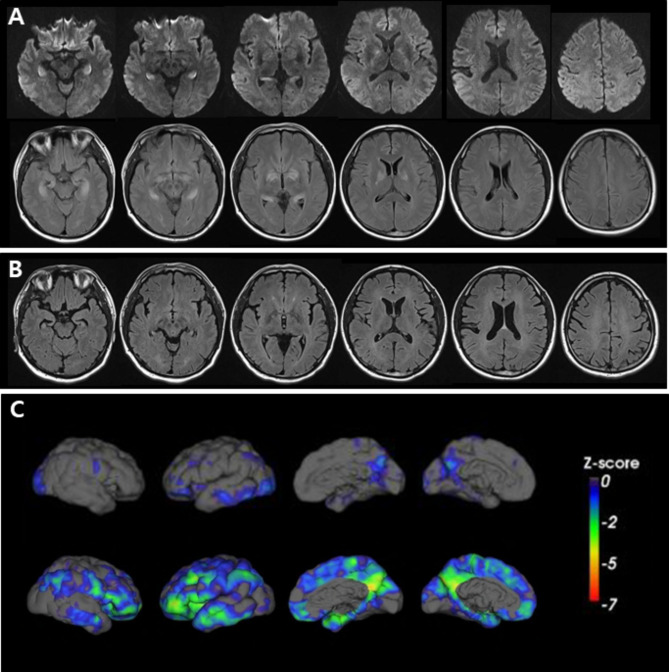
Serial brain magnetic resonance images (MRIs) and [^18^ F]-fluoro-2-deoxy-D-glucose positron emission tomographies (FDG-PETs). (**A**) Initial brain MRIs obtained 10 days after disappearance. Diffusion-weighted images (DWIs) show symmetric high signal intensities in the bilateral hippocampus and globus pallidus (upper row). Fluid-attenuated inversion recovery (FLAIR) images reveal prominent high signal intensities in the same lesions shown on DWIs (lower row). (**B**) Eight-month follow-up FLAIR images show diffuse cortical atrophy with confluent white matter hyperintensities. (**C**) FDG-PET mappings were obtained from the Australian e-Health Research Centre (https://aehrc.csiro.au/). The images were quantified and correlated with 3-D surface image using CapAIBL [20, 21] (https://milxcloud.csiro.au/). To quantify the uptake in PET images, focal uptake values are divided by those of the cerebellum (reference region). After quantification, a Z-score map is created on a 3-D surface image. Predominant precuneus hypometabolism in the initial map (upper row) progresses extensively to the bilateral frontoparietotemporal areas in the 8-month follow-up images (lower row)

**Fig. 2:**
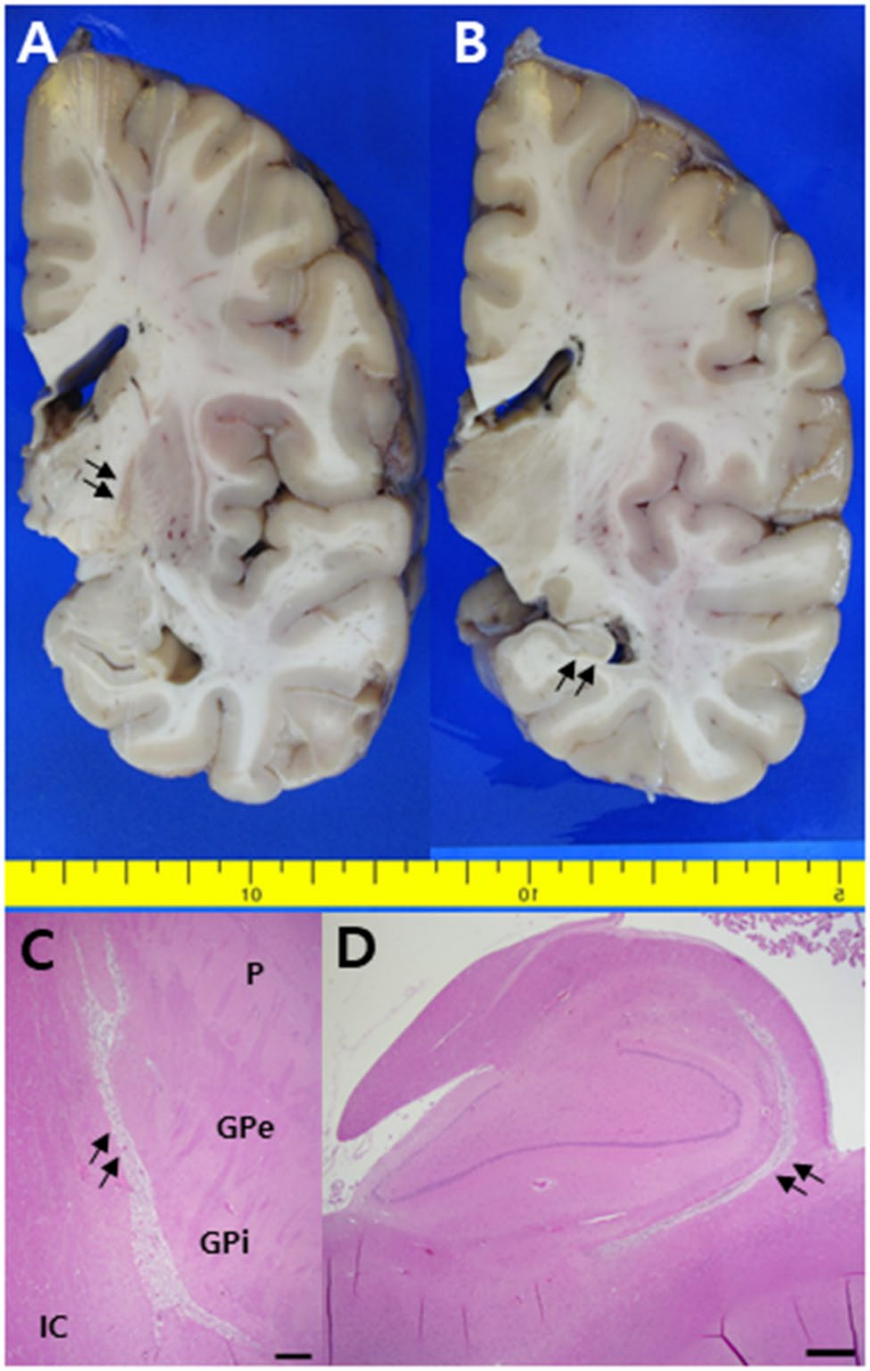
Formalin-fixed coronal sections show necrotic changes in the globus pallidus (**A**, arrows) and atrophic hippocampus (**B**, arrows). Hematoxylin and eosin staining reveals necrotic changes in the corresponding lesions of A (**C**, arrows) and B (**D**, arrows) (scale bar = 250 μm, GPe, globus pallidus externa; GPi, globus pallidus interna; IC, internal capsule; P, putamen)

**Fig. 3:**
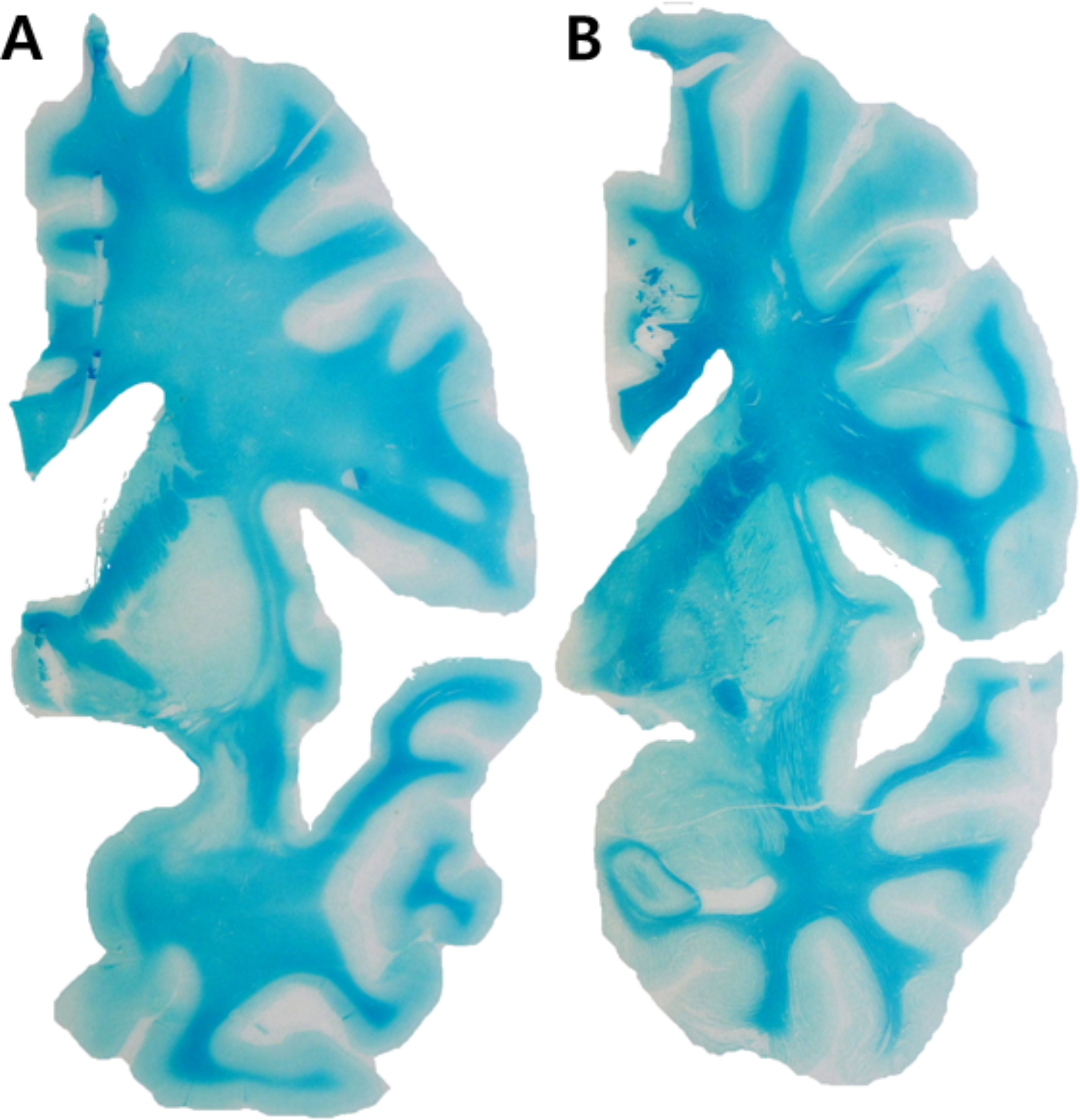
Luxol Fast Blue staining shows diffusely demyelinated frontal white matter of the patient (**A**) in comparison with relatively normal appearing white matter from 77-year-old cognitively unimpaired patient (**B**), which corresponds with white matter hyperintensity seen in eight-month follow up FLAIR images

A 38-year-old man was found in his car with butane-containing cans for a portable cooking gas stove 6 days after disappearance and four days later he was brought to the emergency room because of altered mental status. On arrival, he was mildly drowsy and disoriented to time. He could obey commands; however, his reaction was slow. He showed short-term memory deficits and asked the same questions repeatedly. He exhibited emotional blunting. His vital signs and oxygen saturation were within normal limits. He had isocoric pupils without dilatation. Neurological examination findings were unremarkable. Routine laboratory examination results, including arterial blood gas analyses, cardiac markers, COHb (0.1%), and metHb (0.6%) were all normal. There were no abnormalities on the electrocardiogram or echocardiogram. A mini-mental state examination score was 24 of 30. T2-weighted and fluid-attenuated inversion recovery brain MR images showed symmetric high signal intensities involving the bilateral hippocampus and globus pallidus, which were also detected in diffusion-weighted images (Fig. 1A). The apparent diffusion coefficient values were also high. FDG-PET revealed glucose hypometabolism in the bilateral precuneus, occipital, and left temporal areas (Fig. 1C, upper row). An electroencephalogram showed intermittent theta to delta slowing. On the third day of admission, his mental status recovered from drowsiness to alertness. Detailed neuropsychological test results showed deficits in verbal and visual memory and frontal executive function (Table 1). Six days after admission, he remained stable without clinical aggravation. At the 8-month follow-up neuropsychological evaluation, he showed mild improvement compared with his initial performance but still significant deficits in memory and frontal functions in comparison with education-matched, 45-year-old cognitively normal controls (Table 1). However, abnormal behaviors such as irritability, obsession with food, and lethargy became more prominent. Follow-up brain MRIs showed diffuse cortical atrophy with white matter hyperintensities (Fig. 1B). Follow-up FDG-PET revealed glucose hypometabolism more extensively involving the bilateral frontoparietotemporal areas (worse on the left) than the previous one (Fig. 1C, lower row). Two months after the second evaluation, he died unexpectedly, and a brain autopsy was performed. Grossly, there was no definite cortical atrophy. However, necrosis and cavitary lesions in the globus pallidus (Fig. 2A) and atrophy in the hippocampus (Fig. 2B) were detected. Microscopically, hematoxylin and eosin staining revealed severe gliosis, necrotic changes, and neuronal loss in the globus pallidus and hippocampus (Fig. 2C and D). Luxol fast blue staining revealed widespread demyelination of the subcortical white matter (Fig. 3). Immunohistochemical staining for tau, β-amyloid, TAR DNA binding protein, and α-synuclein revealed no abnormalities in immunoreactivity.

**Table 1:** Results of the neuropsychological tests

Neuropsychological test	Results	
	**1st**	**2nd**
Digit span: forward/backward	6/3	8/5
Letter cancellation, vigilance test	NL	NL
Fluency	Fluent	Fluent
Comprehension/repetition/reading/writing	NL/NL/NL/NL	NL/NL/NL/NL
K-BNT	51/60	49/60*
Calculation	11/12	11/12
Finger naming/R.-L. orientation/body part identification/praxis	NL/NL/NL/NL	NL/NL/NL/NL
Interlocking pentagon	NL	NL
Rey Complex Figure Test copy	36/36	33/36
SVLT free recall/delayed recall	8(2 + 3 + 3)*/0*	13(5 + 3 + 5)*/4*
RCFT immediate recall, 20-min delayed recall	0/36*, 0/36*	3/36*, 0/36*
Semantic word fluency: animals/supermarket items	4/6*	6/5*
Phonemic word fluency: ㄱ/ㅅ/ㅇ	3/1/4*	5/4/5*
Stroop test: word/color	112/45*	112/78*
Trail making test: part A/part B	17s/30s	12s/25s
Digit symbol coding	46*	57*
MMSE	18/30	23/30
CGA-NPI	16/144	37/144
FBI	26/72	39/72

CGA-NPI, Caregiver-Administered Neuropsychiatric Inventory; FBI, Frontal Behavioral Inventory; K-BNT, Korean version of the Boston Naming Test; MMSE, Mini-Mental State Examination; L, Left; RCFT, Rey Complex Figure Test; R, Right; s, seconds; SVLT, Seoul Verbal Learning Test; NL, within normal limit; *, lower to 1 standard deviation of education-matched, 45-year-old cognitively normal controls.

## Discussion and conclusions

Butane is a highly lipophilic and volatile substance frequently used as a fuel source for cooking at home and camping in Korea. Although fatal cardiac arrhythmia due to myocardial sensitization to catecholamine after butane intoxication is well documented, little is known about the potential mechanism of encephalopathy associated butane inhalation [1, 2, 9]. Kile et al. reported a 16-year-old male with acute butane encephalopathy associated with bithalamic lesions. They suggested that bithalamic injury could have been attributed to direct butane toxicity (toxic-metabolic encephalopathy) rather than anoxic or hypoxic brain injury resulting from asphyxia by butane displacing oxygen [7]. Thalamic injury has been described in other types of toxic-metabolic encephalopathies (7) but less so than other lesions, such as the globus pallidus in anoxic or hypoxic encephalopathy (10). Previous reports on butane encephalopathy described brain MRI findings from normal to various brain lesions, including the thalamus, putamen, cerebellum, insula, and occipital cortex [1, 2, 7, 11–14]. However, as in our case, bilateral hippocampal and globus pallidal lesions have not yet been reported. One case of toxic encephalopathy revealed bilateral hippocampal and white matter damage; however, the toxic substance was propane gas, not butane, although both were liquefied petroleum gases [6]. The globus pallidus is most commonly affected in anoxic–ischemic encephalopathy due to carbon monoxide (CO) poisoning because of its high metabolic demands and weak vascular perfusion [10]. The hippocampus may also be involved in CO poisoning, as it is the region most vulnerable to hypoxic–ischemic injury [15, 16]. Therefore, based on radiological findings, we could assume that hypoxic–ischemic encephalopathy developed after exposure to butane. As the patient did not have any cardiac problems, hypoxic–ischemic events may have resulted from asphyxia after butane inhalation. Indeed, it may be doubtful why restricted diffusion along with high ADC values in predilection areas was observed in our case. There has been a report that transient vasogenic edema can be occurred after acute CO exposure [10]. Alternatively, it could be explained that the initial low ADC values reflecting cytotoxic edema gradually increased (vasogenic edema) during the 4 or 10-day interval from the patient’s exposure to butane to taking the initial MRI. No previous reports of butane encephalopathy have described serial MRI findings. However, diffuse cortical atrophy at the 8-month follow-up MRIs in our case corresponded well to those in prior studies addressing marked atrophic changes occurring within 6 months of CO exposure, and these changes were attributed to neuronal loss and necrosis in the acute stage [10, 17]. Furthermore, diffuse white matter changes on follow-up MRIs are consistent with those detected in delayed posthypoxic encephalopathy, which is evidence of extensive demyelination [18]. The radiological findings after representative toxic encephalopathy or hypoxic-ischemic injury are summarized in Table 2.

**Table 2:** Comparison of radiological findings between representative toxic and hypoxic-ischemic encephalopathies

	Toxic encephalopathy	Acute hypoxic-ischemic encephalopathy	Delayed hypoxic-ischemic encephalopathy
Etiology	Drugs of abuse (e.g. Butane, Heroin)	Cardiac arrest Acute respiratory insufficiency Other toxic causes (e.g., CO)	Cardiorespiratory compromise Other toxic causes
MRI findings	- High FLAIR or T2 signal intensities in the thalamus, basal ganglia, cerebellum, or insula (Butane intoxication) - High signal changes in the putamen, and occipital and frontal lobes in DWI (Butane intoxication) - Symmetrically increased T2 and FLAIR signal intensity of the cerebellar and posterior cerebral white matter, posterior limb of the internal capsule with sparing of the anterior limb of the internal capsule and dentate nuclei (Heroin inhalation)	- Diffuse cortical injury on DWI - Border zone distribution of ischemia - Increased T2 and FLAIR signal intensity in the globus pallidus along with corresponding diffusion restriction - Much less commonly increased T2 and FLAIR signal intensity in the caudate, putamen, thalamus, hippocampus, cerebellum, and brain stem than that in the globus pallidus	- High T2-FLAIR signal intensity in the periventricular white matter and centrum semiovale sparing the cerebellum and the brainstem tracts
Reference	[2, 7, 11, 22]	[10, 23]	[24]

MRI, magnetic resonance imaging; FLAIR, Fluid-attenuated inversion recovery; DWI, Diffusion weighted image; CO, Carbon Monoxide

Regarding FDG-PET findings, initially predominant glucose hypometabolism in the precuneus progressed to extensive frontoparietotemporal hypometabolism at the 8-month follow-up. Interestingly, the initial finding of selective precuneus hypometabolism partially corresponds with previous research demonstrating reduced functional connectivity between the hippocampus and precuneus in the early stage of Alzheimer’s disease with hippocampal atrophy and structurally unaffected precuneus [19]. Therefore, selective impaired memory in the first neuropsychological test in our case was generally consistent with the initial brain injury pattern. However, his performance remained generally unchanged during the follow-up test and was not correlated with the widely distributed glucose hypometabolism attributed to diffuse cortical atrophy with white matter hyperintensities on follow-up images. Only one previous report has described cognitive performance in detail after butane inhalation. Woods et al. reported a 14-year-old girl with severe verbal and nonverbal declarative memory impairment with normal brain MRI findings after successful resuscitation following ventricular fibrillation arrest [13]. In contrast to our case, the patient’s cognitive function showed improvement or returned to normal at 3-month follow-up [12].

Lastly, autopsy results showing necrosis of the globus pallidus and extensive white matter demyelination confirmed those findings on brain MRIs.

Our case is of particularly interest because, to the best of our knowledge, this is the first report describing serial structural and functional images with autopsy findings associated with butane encephalopathy. However, it should also be noted that these neuroimaging and pathological findings may not be specific for butane encephalopathy but may be present in any patient with hypoxic–ischemic encephalopathy, regardless of any etiological volatile substance.

## Data Availability

Data sharing is not applicable to this article as no datasets were generated or analysed during the current study.
